# Integrating impact of FSH isoforms, androgens and inhibin-B on follicular development based on a two-phase model of the follicular phase

**DOI:** 10.1186/s12958-025-01464-2

**Published:** 2025-09-30

**Authors:** Claus Yding Andersen

**Affiliations:** 1 The Fertility Clinic, Copenhagen University Hospital Herlev, Herlev, 2730 Denmark; 2 Institute of Clinical Medicine, Faculty of Health and Medical Sciences, University of Copenhagen, Copenhagen, Denmark; 3 Department of Urology, Copenhagen University Hospital - Herlev and Gentofte Hospital, Herlev, Denmark

**Keywords:** Androgens, Follicular development, FSH isoforms, Inhibin-B, Paracrine signalling

## Abstract

**Background:**

Follicle Stimulating Hormone (FSH) is central to follicular growth during the menstrual cycle. It is secreted in multiple isoforms that differ in glycosylation, bioactivity, and half-life. Acidic isoforms dominate early in the follicular phase, while less acidic forms rise closer to ovulation caused by increasing oestradiol. Though well studied in vitro, the distinct physiological roles of these isoforms in vivo are not fully understood.

**Methods:**

We conducted a narrative review of published literature focusing on FSH isoforms composition, glycosylation patterns, and their functional roles during the human menstrual cycle, with emphasis on isoform-specific actions in follicular development.

**Main findings:**

It is proposed that acidic FSH isoforms primarily support early follicular grow by stimulating inhibin-B production, which enhance androgen synthesis in synergy with LH. These androgens, in turn, increase FSH receptor expression in granulosa cells, promoting follicle sensitivity. In the later follicular phase, less acidic isoforms support final follicular maturation by upregulating aromatase and LH receptor expression in granulosa cells, facilitating the shift from androgen to oestrogen production.

**Conclusion:**

The sequential dominance of FSH isoforms appears to guide distinct stages of follicular development. Understanding this temporal regulation may lead to improvements in ovarian stimulation strategies and enhance outcomes in assisted reproduction.

## Introduction

Follicle stimulating hormone (FSH) is the single most important hormone in regulation of ovarian follicle growth and development. FSH exerts a multitude of functions on follicular granulosa cells (GC), which in women are the main cells expressing functional receptors (FSHR). During the several months long development from the early stages of folliculogenesis to the preovulatory stage, it has been estimated that FSH induces up to 200 different signals in GCs [1]. Furthermore, FSH is vital in the last stages of development during the follicular phase of the menstrual cycle, where follicles grow from around 2-5 mm in diameter to the around 20 mm right before ovulation. In addition to FSHR expression on GCs, FSHR has also recently been reported to be expressed on other cell-types such as pluripotent stem cells and cancer cells in multiple organs including the ovaries [2–6]. In addition, FSH has been shown to stimulate proliferation of stem cells and germ cell nests formation even in an adult ovary [2–6].

The concentration of FSH shows a characteristic pattern in the follicular phase of the menstrual cycle with increasing concentrations from around 2-4 IU/L at start of menstruation peaking around cycle day 6-8 at a level of around 6-9 IU/L. In the second half of the follicular phase concentrations of FSH gradually return to around 3-5 IU/L shortly prior to ovulation, during which FSH levels reach 12-15 IU/L [7]. This dynamic FSH profile ensures selection of a single dominant follicle, while also affecting ovarian steroidogenesis including the production of androgens by the theca cells (TCs) and oestradiol and progesterone by the GCs. Given the extensive signalling complexity of FSH in GCs and the effects on TCs via paracrine stimulation, a gap remains in understanding how the natural variation of the different FSH isoforms interact and influence follicular development especially in women.

This review aims to contextualize the distinct FSH fluctuations, FSH isoforms and androgen and oestrogen production during the follicular phase within their physiological framework.

## Material and methods

This review was based on a comprehensive literature search using PubMed and Web of Science to identify original studies, reviews, and relevant articles on FSH isoforms, glycosylation, and ovarian follicular development in humans. Priority was given to peer-reviewed publications with mechanistic, clinical, and translational relevance. Studies involving animal models were considered where appropriate to complement human data. Reference lists of key articles were also screened for additional relevant literature.

## Results

### The follicular phase of the menstrual cycle has different FSH requirements in the first and the second half

**Fig. 1:**
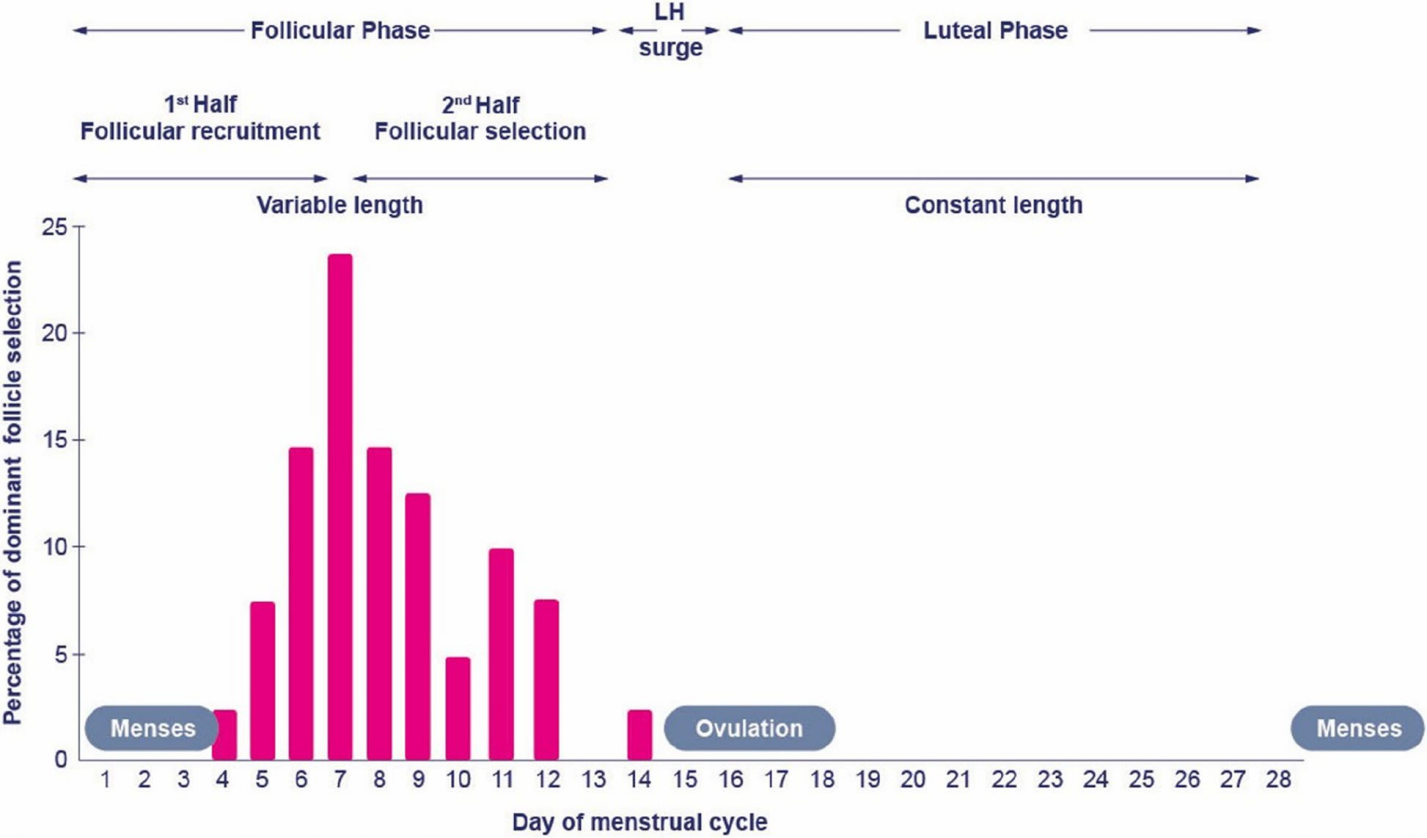
The menstrual cycle consists of three phases; the follicular phase, the LH surge, and the luteal phase. The follicular phase can be divided into two parts: the first half, lasting until approximately cycle day 7, and the second half, spanning from follicular selection for dominance to the initiation of the mid-cycle LH surge. The cycle day at which the dominant follicle can be identified is depicted in pink as obtained from [9]

Follicular development and hormonal dynamics during the follicular phase have been proposed to consist of two distinct stages: an initial stage lasting approximately seven days, followed by a second stage that extends until ovulation, typically lasting 5–7 days first introduced by S. Yen almost half a century ago [8, 9] (Fig. 1). The first stage encompasses follicular recruitment, during which a cohort of follicles is stimulated to growth and development beginning with a diameter of 2-4 mm, whereas the second stage is characterized by the growth of the one selected follicle, culminating with a diameter of around 20 mm at ovulation. The transition between these stages is marked by the selection of the dominant follicle, which occurs between cycle days 6–8 in most women (Fig. 1) [9].

The physiological roles of FSH and LH differ markedly across these two stages of the menstrual cycle. This review focuses specifically on the effects of FSH isoforms and the roles of inhibin B and androgens in women. Inhibin B and inhibin A are members of the TGF-β superfamily and are produced almost exclusively by GCs in response to FSH stimulation [10]. Both inhibin’s accumulate in follicular fluid (FF) at concentrations approximately a thousand-fold higher than in circulation (ng/mL vs. pg/mL), where they exert a range of paracrine effects within the follicle (as discussed below). When secreted into the bloodstream, inhibin’s act in an endocrine manner by suppressing pituitary FSH secretion [10].

### FSH isoforms

**Fig. 2:**
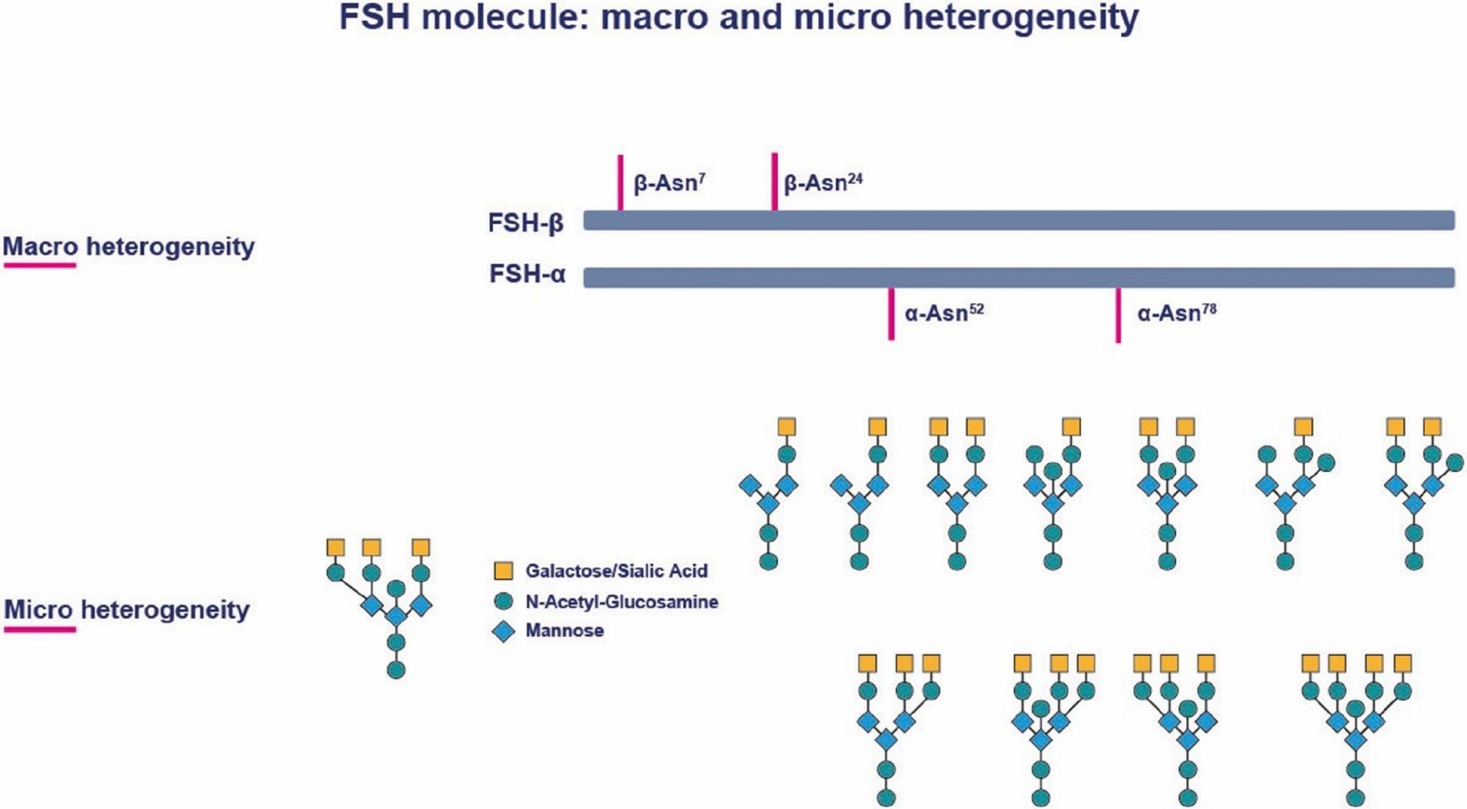
The FSH molecule with peptide backbone consisting of an α and β chain plus four potential glycosylation cites. Definition of macro and micro heterogeneity

The pituitary synthesizes FSH which consists of two non-covalently linked peptide chains: an α-chain and a β-chain. The β-chain is unique to FSH and determines its specific biological function. FSH has four asparagine-linked glycosylation sites—two on each of its peptide chains—where oligosaccharides can attach [11]. The heterogeneity of FSH arises from 1) whether these glycosylation sites have attached glycans or not, and 2) from variations in the specific sugar residues involved in the individual oligosaccharides (Fig. 2). Importantly, the amino acid sequence of FSH remains consistent across all types of human FSH, except in rare cases of mutations and polymorphisms. This uniformity applies regardless of whether FSH is synthesized by the pituitary gland, produced in transfected animal or human cell lines, or derived from human biological fluids like for instance urine.

FSH isoforms are classified based on two types of heterogeneity: macro- and micro-heterogeneity (Fig. 2).

#### Macro-heterogeneity

Macro-heterogeneity refers to the presence or absence of oligosaccharides at FSH’s four potential N-linked glycosylation sites. While the two glycosylation sites on the α-chain are constituently glycosylated, the sites on the β-chain may or may not have attached sugar residues. The β-chain glycosylation sites are located at the amino acid asparagine in positions 7 and 24. The oligosaccharides affect the molecular weight (Mw) of FSH, as observed in Western blot analysis: Thus, if both glycosylation sites are occupied the Mw will be 24 KDa and the FSH isoform will be designated FSH^24^, if only one of the two sites is glycosylated the Mw will be lower, FSH^21^ and FSH^18^, respectively. If neither of the two sites are occupied, leaving only the α-chain double glycosylated, it is determined as FSH^15^, which remarkably, is not secreted by the pituitary [11] (Fig. 2).

These differences in glycosylation impact the 3-dimensional structure and biological activity of each FSH isoform. Glycosylation also influences the hormone’s circulatory half-life, with FSH^24^ exhibiting a longer half-life compared to FSH^21^ and FSH^18^. The presence of oligosaccharides at asparagine 24 reduces FSHR binding and signal transduction, while FSH^21^ and FSH^18^ demonstrate higher FSHR affinity and in vitro biological activity. However, the precise effects on intracellular signalling pathways are still being investigated [11].

#### Microheterogeneity

Although the peptide backbone of FSH remains unchanged, significant variability exists in the oligosaccharides attached to its glycosylation sites. This variability arises from differences in sugar composition and the complexity of the carbohydrate structures, which can range from single-branched to di-, tri-, or even tetra-branched formations. Additionally, each branch of the residue may terminate with a negatively charged sialic acid residue (Fig. 2) [12–14].

As a result, each FSH molecule is characterized by a specific number of negatively charged sialic residues that affects their isoelectric point (pI), which define the pH at which the molecule has no net charge. Thus, FSH molecules exhibit as a broad spectrum of isoforms, differing in their carbohydrate composition, complexity, and isoelectric points (pI). More acidic FSH isoforms contain a higher number of sialic acid residues and exhibit more extensive branching patterns, while less-acidic isoforms have fewer sialic acid residues and simpler carbohydrate structures although FSH preparations usually contains both acidic and less acidic isoforms but to a varying degree [15]. This charge variation allows FSH isoforms to be separated by electrophoresis, with most isoforms having a pI between 4.5 and 5.0, though the range extends from approximately 3.5 to 7.4 [12–15].

The exogenous FSH preparations used for ovarian stimulation during assisted reproduction differ in their FSH isoform composition depending on the source of FSH and the purification process implying that they may potentially influence follicular response differently. Urinary-derived FSH is obtained from postmenopausal women, who only produce low levels of oestradiol and consequently the pituitary predominantly secretes acidic FSH isoforms, while recombinant human FSH in contrast has been engineered to contain a less acidic isoform profile [15]. Thus, commercial gonadotropin preparations all contain FSH isoforms spanning a range of different pI’s but preparations based on urine contains a higher fraction of acidic isoforms, while recombinant products predominantly contain less acidic isoforms. A recent review found that a slight increase in the number of follicles obtained in clinical trials comparing recombinant and urine derived products could be related to other conditions not related to the FSH isoform composition such as the dose administered and concluded that appropriate trials were warranted [16].

### FSH isoforms during the follicular phase

**Fig. 3:**
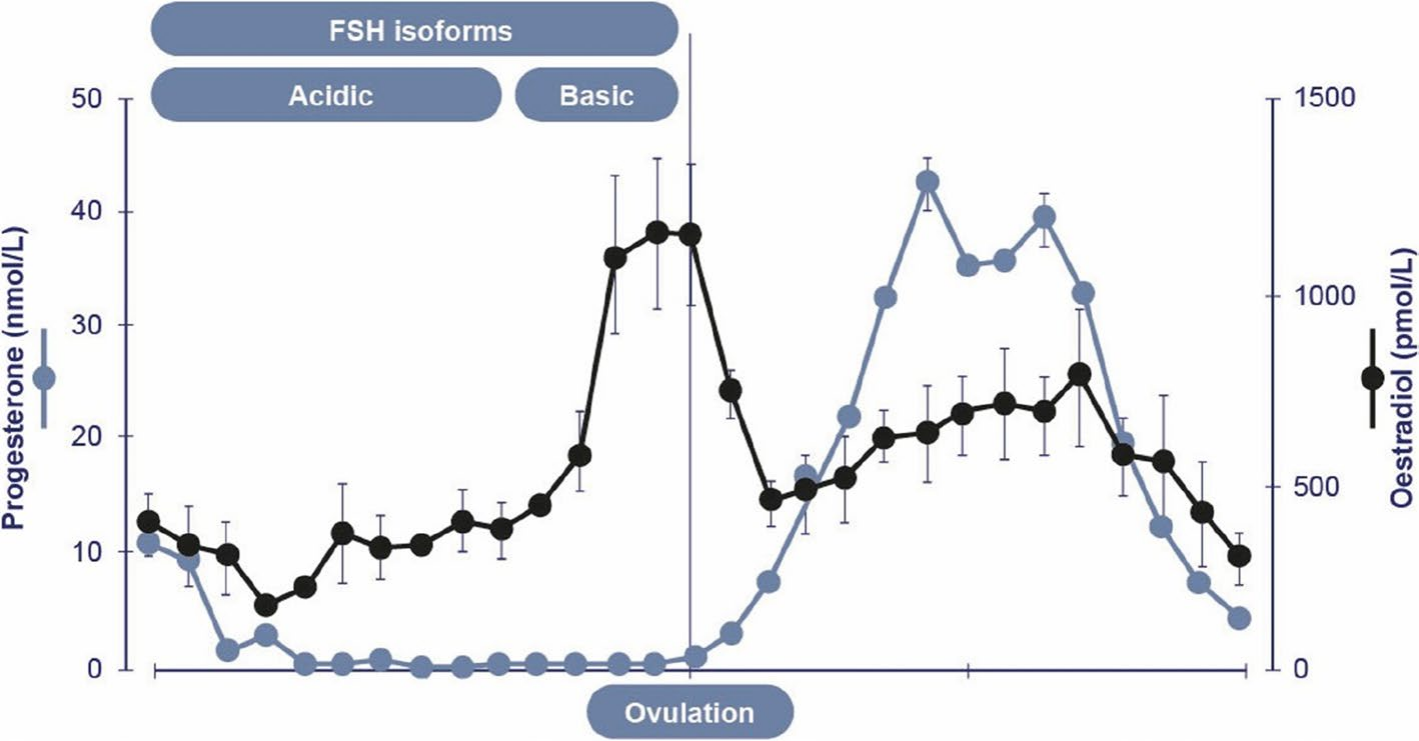
Concentrations of oestradiol and progesterone throughout the natural menstrual cycle with distribution of the majority of FSH isoforms during the follicular phase (Data combined from [7, 18]

Changes in the distribution of circulating FSH isoforms occur during the follicular phase determined mainly by the sialic acid content of the attached oligosaccharides and the concentration of oestradiol [12, 17–19] (Fig. 3). FSH isoforms with a higher sialic acid content (i.e., acidic isoforms) are metabolized more slowly by the liver, resulting in a longer circulatory half-life [12]. In contrast, less-acidic isoforms, which contain fewer sialic acid residues, are cleared more rapidly from circulation. During the follicular phase, the composition of FSH isoforms mirrors the concentrations of oestradiol with acidic isoforms predominating during the early to mid-follicular phase. As ovulation approaches and oestradiol increases, however, there is a shift towards less-acidic isoforms [17–20] (Fig. 3).

Oestradiol directly affects downregulation of pituitary glycosyltransferases, including 2,3 α-sialyltransferase (an enzyme responsible for catalysing integration of sialic acid residues into oligosaccharides) [21]. Conversely, during conditions of low oestradiol levels, such as in postmenopausal women, the secretion of more acidic isoforms predominates. Administration of oestradiol to postmenopausal women induces a shift towards the release of less-acidic FSH isoforms, thereby mimicking the hormonal environment of the follicular phase [22].

Thus, the physiological relevance of the changing FSH isoform profile during the follicular phase may represent an important mechanism to provide optimal stimulation to the follicle at different stages of development [23–26]. The increased presence of less-acidic FSH isoforms likely provides potent, short-lived stimulation for dynamic events such as ovulation [27], while the more acidic isoforms are essential for the recruitment and growth of follicles during the early to mid-follicular phase.

### In vitro bioactivity of FSH isoforms

**Fig. 4:**
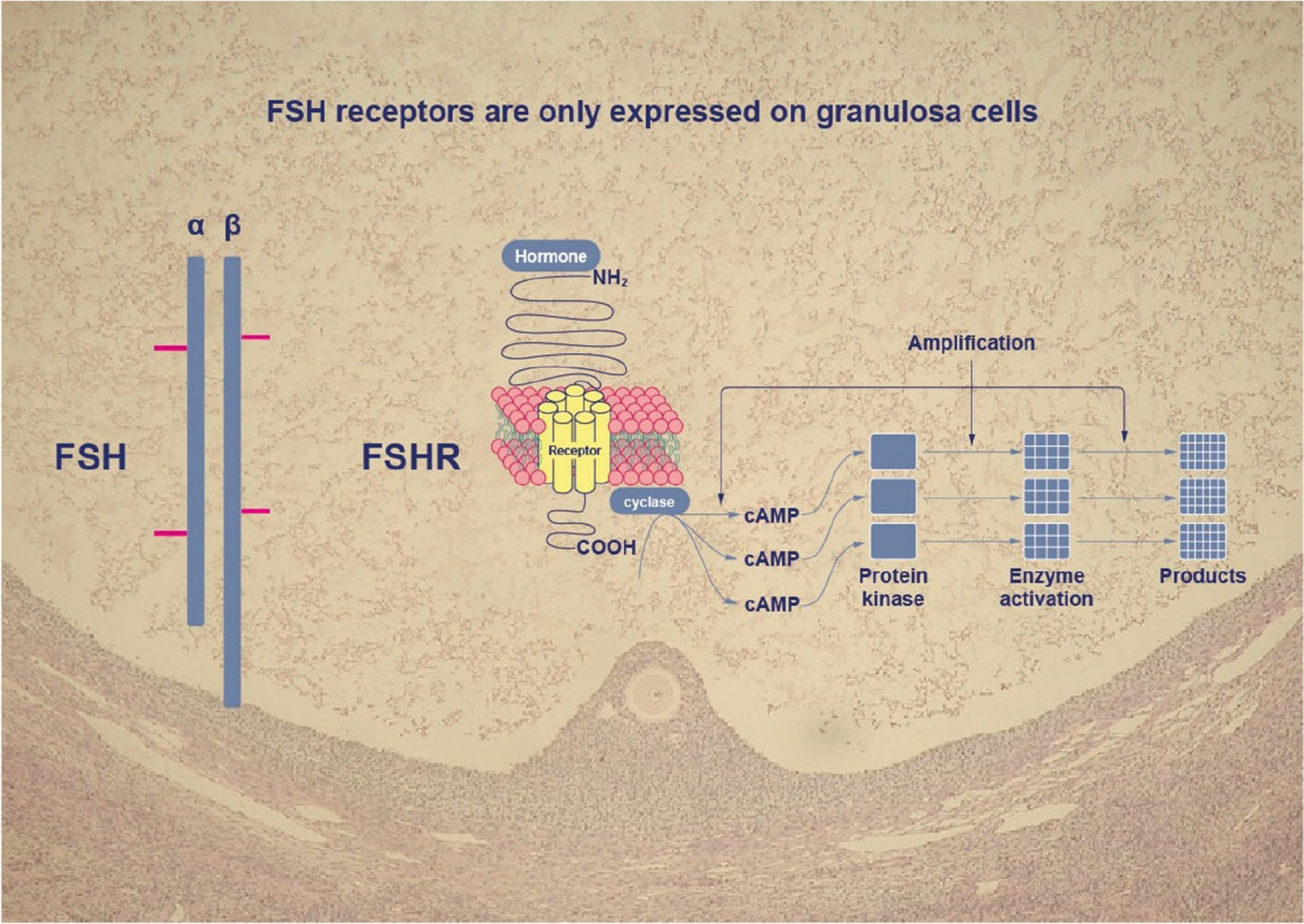
FSH receptors are mainly expressed on granulosa cells and result in a multitude of intracellular signals and activation of various signalling pathways – graphics overlayed with a histological section of a human antral follicle 8 mm in diameter

Whereas immunoassays have been used to provide a measure for the concentration and mass of FSH, the functional aspects of the FSH ISOFORMS are frequently measured by cell-based assays [25, 28–30]. The different FSH isoforms all bind to and activate the single FSH receptor (FSHR) type, which is a G-protein coupled receptor that uses cAMP and other intracellular messengers upon activation (Fig. 4).

Assays used to monitor the biological activity of FSH and FSH isoforms includes:

1. Initially the rat GC or the Sertoli cell aromatization bioassays were used [12] to show that cAMP, oestrogens and tissue-type plasminogen activator, as well as CYP19A1 cytochrome P450 aromatase activity were all significantly up-regulated by less acidic FSH isoforms as compared to acidic isoforms suggesting a more effective triggering of G_s_-mediated intracellular signalling [31, 32].
2. In contrast, the acidic FSH isoforms significantly augmented α-inhibin mRNA production as compared to less acid FSH isoforms in rats [33]. On a protein level this was in vitro confirmed by Loreti and co-workers [34], who in addition found that rat GCs produced more inhibin-A, when stimulated with less acidic FSH isoforms.
3. In a mouse assay less acidic human FSH isoforms induced resumption of meiosis in cumulus oocyte complexes significantly more efficiently (twice more effective) than acidic isoforms [28].
4. Using recombinant human FSH in vitro growth of isolated mouse preantral follicles were significantly increased by less acidic FSH isoforms as compared to acidic isoforms. Furthermore, the less acidic isoforms also had a higher effect on oestradiol production and antral follicle formation [25, 35].

Although most of these studies were performed more than two decades ago, the results are still considered valid and less acidic isoforms are generally perceived as more potent than the acidic isoforms [14, 36]. The current concept is that the less acidic FSH isoforms possesses a higher receptor affinity and higher FSHR binding. However, the results from the inhibin-B production obtained in rats showed that it cannot be ruled out that particular effects of the isoforms may relate to diverse abilities of the different oligosaccharides to interact with the FSHR and stimulate different downstream signal transduction pathways.

### FSH as a biased agonist

**Fig. 5:**
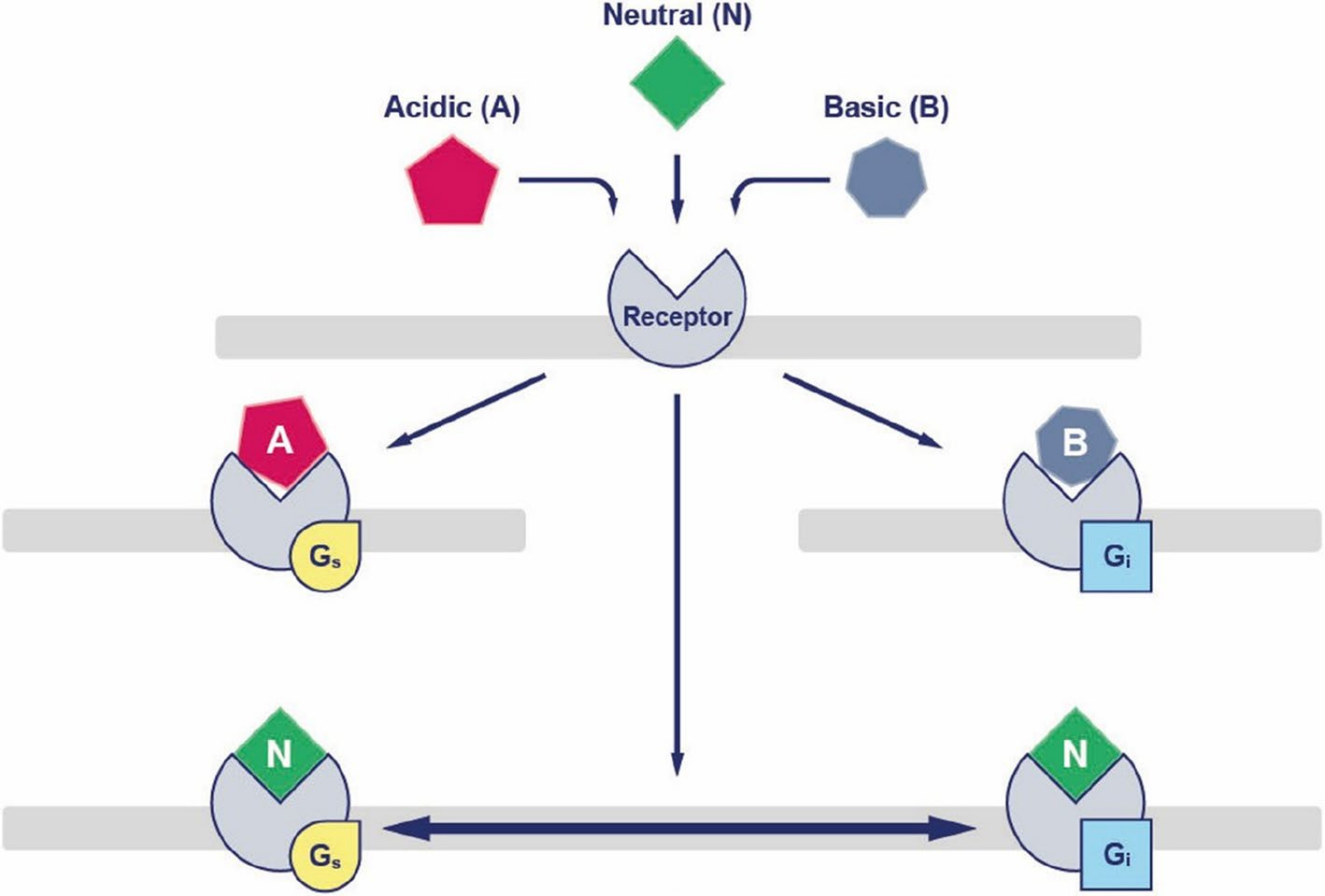
Isoforms of FSH exert separate signalling properties that allow for an excessive number of signals to be transduced effectively via the FSH receptor. This classify FSH isoforms as biased agonists

As described, FSH triggers various intracellular signalling pathways and different FSH isoforms likely interact with FSHR in distinct ways, triggering specific signalling responses. Consequently, FSH (along with LH and human chorionic gonadotropin (hCG)) has been termed biased agonists (Fig. 5) [37].

This emphasizes that the natural fluctuations of FSH isoforms is likely to have diverse physiological functions and this concept is particularly relevant given that pituitary FSH secretion and isoform profile fluctuates throughout the follicular phase and is governed by circulatory concentrations of oestradiol (as discussed above).

Ovarian stimulation invariably leads to supraphysiological concentrations of oestradiol and this aspect is hypothesized to impact on the currently most frequently used ovarian stimulation protocol, which includes the use GnRH-antagonists to avoid a premature LH release. These GnRH antagonists have been developed to reduce endogenous FSH production to only a limited extent, whereas LH suppression is more pronounced, effectively preventing premature LH release [38, 39].

Consequently, follicles are therefore exposed to a combination of endogenous and exogenous FSH isoforms. Notably, the supraphysiological oestradiol concentrations achieved during ovarian stimulation likely alter pituitary glycosylation patterns of FSH, favouring release of less acidic isoforms. In addition to differences in plasma half-life follicles are exposed to an ever-changing in vivo FSH isoform profile, complicating the assessment of its clinical relevance. Despite clear qualitative and quantitative differences in GC signalling induced by different FSH isoforms in vitro, predicting their precise impact on ovarian stimulation outcomes remains challenging.

### Androgens in follicular development during the follicular phase of the menstrual cycle

Androgens exert their effects via the androgen receptor (AR) and play critical roles in normal follicular development and overall fertility with expression of the AR on both TCs and GCs [40–42].

While abnormal levels of androgens as for instance in women with polycystic ovaries affects and may attenuate follicular development, normal physiological levels of androgens support follicular growth and development prior to follicular selection potentially via enhanced FSHR expression [43, 44].

Androgen Receptors are expressed in multiple organs throughout the body affecting multiple functions [44], however, one study in mice in which a GC specific knock-out of AR was developed, showed a direct effect on the ovaries with compromised fertility and reduced ability of follicular growth [45] and suggests central functions of androgens in follicular development also in humans.

**Fig. 6:**
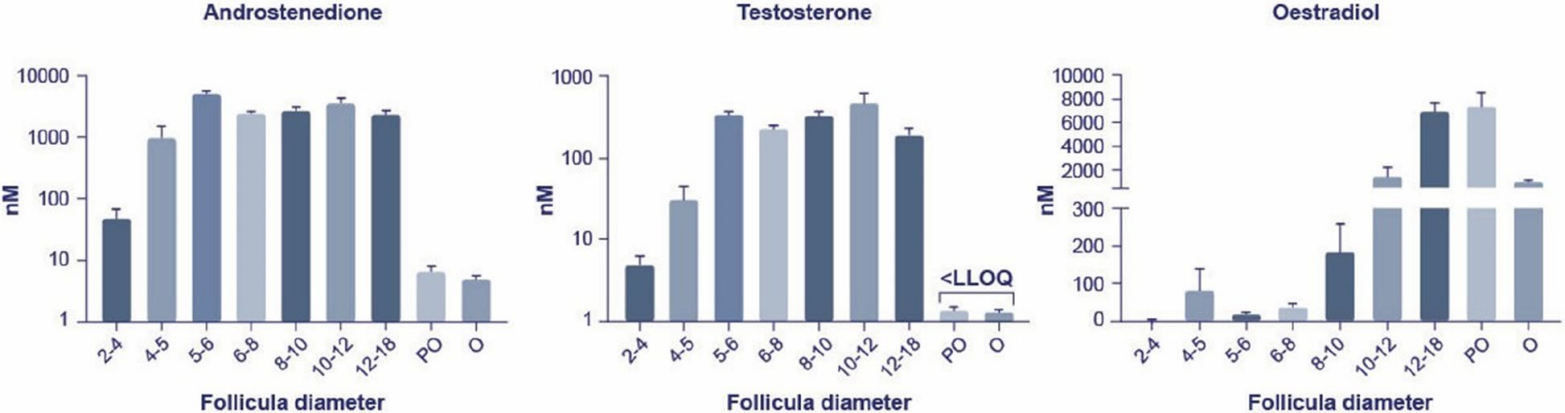
Concentrations of androstenedione, testosterone and oestradiol in follicular fluid from normal human follicles in relation to diameter measured by liquid chromatography-tandem mass spectrometry as described previously [48]

It is important to note that the concentration of the two primary androgens — testosterone and androstenedione — differs significantly between circulation and follicular fluid (FF) of small antral follicles in humans. In circulation, the concentrations of androstenedione range from 2 to 8 nM, and testosterone from 0.6 to 2.5 nM [46, 47]. In contrast, FF concentrations in small antral follicles are much higher, reaching 2000–5000 nM for androstenedione and 250–400 nM for testosterone [10, 42]. However, both these concentrations decrease markedly in preovulatory follicles (Fig. 6) [48, 49].

Thus, in the first half of the follicular phase high intrafollicular androgen concentrations promote FSHR expression, FSH sensitivity and follicular recruitment. In contrast, androgens are more than halved after selection in second half of the follicular phase and almost disappear after initiation of the midcycle surge of gonadotropins [43, 50]. Thus, if androgens remain high in the second half of the follicular phase this most likely reflects a reduced expression of CYP19A1 and attenuated ability to aromatise androgens into oestradiol indicating less viable GCs and reduced follicle health. In order to induce CYP19A1 expression, less acidic FSH isoforms probably play an important role in the second half of the follicular phase. Alternatively, a continued high concentration of androgens would result in signal transduction via the AR that maintain to be expressed on GCs, albeit at lower levels [34, 36], and could constitute another explanation for negative effects of androgens in the second half of the follicular phase. Thus, these effects potentially taking place in the second half of the follicular phase essentially illustrate the flip side to the positive effects of androgens in the first half of the follicular phase.

Both basic science and clinical data support a beneficial role of androgens during the recruitment phase of follicular development [40, 43]. Studies conducted by Carolin Bondy’s group at NIH, Bethesda, more than 25 years ago demonstrated that exogenous testosterone administration in female monkeys led to increased follicular recruitment, enhanced FSHR expression, and augmented GC proliferation markers [51, 52]. Furthermore, this androgen treatment resulted in significant, 3-4-fold increases in IGF-I mRNA concentration and in IGF-I receptor mRNA in GCs and TCs especially in small growing follicles (≤1 mm diameter) [53]. However, these studies used high doses of testosterone, which will induce masculinising effects in women and are incompatible with clinical use in women (administration of around 5 mg testosterone daily (≈ 0.1 mg/kg) lead to concentrations above the physiological levels [54, 55], while the primates in the above-mentioned studies received either 0.4 or 4 mg/kg [51–53]. Yet, as indicated above, the concentrations of androgens within follicles are a more than a hundred times higher than in circulation and illustrates that augmenting only the intrafollicular concentrations may locally cause the desired effects.

**Fig. 7:**
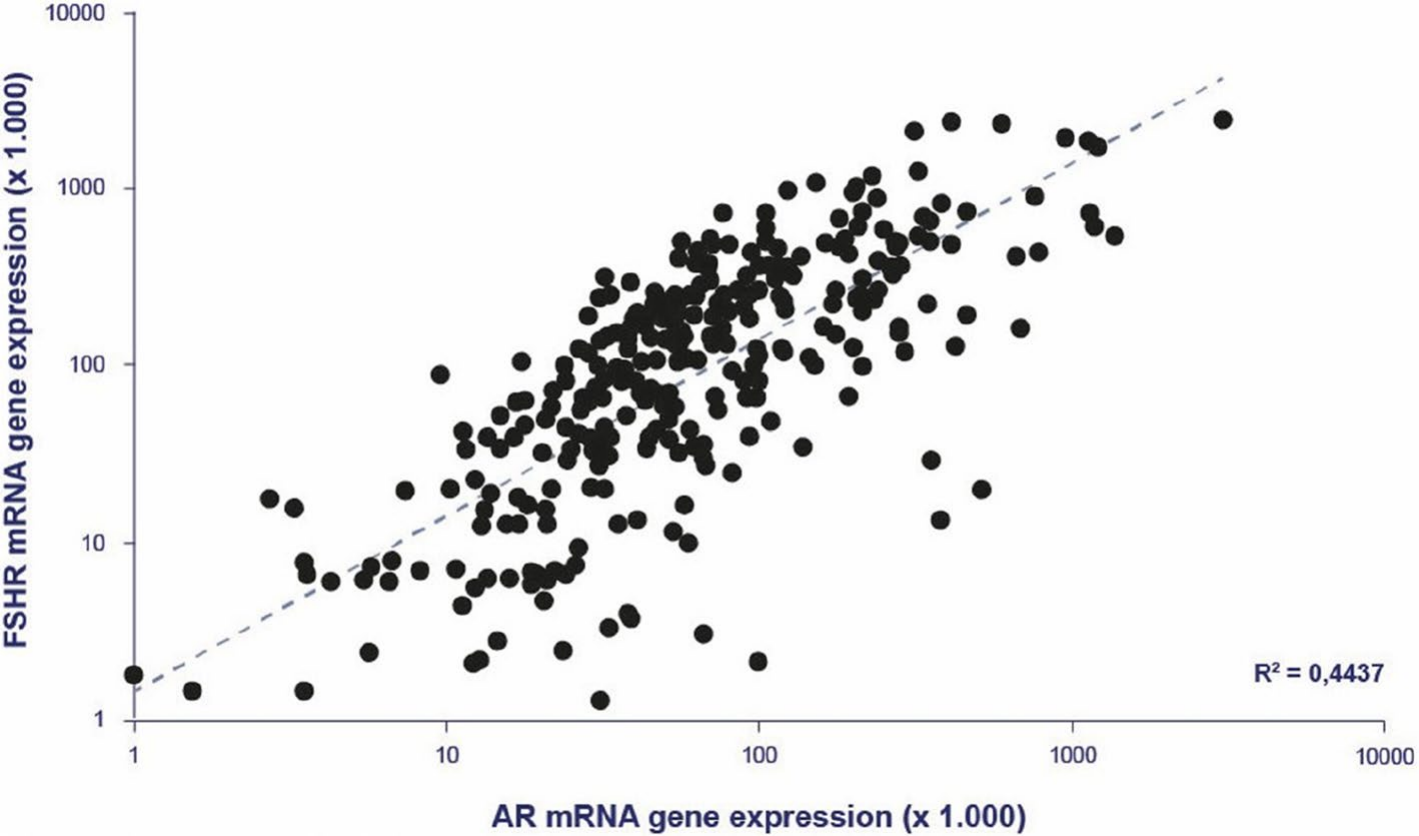
Granulosa cells from human small antral follicles: correlation between *FSH receptor* and *Androgen receptor* mRNA expression levels in 316 individual follicles. Spearman correlation coefficient: *R*^2^ = 0.44 (*P* < 0.001). Data are based on reference [10], with an expanded sample size obtained using the same q-PCR gene expression methods described therein

In a study on human small antral follicles (3–9 mm in diameter), the gene expression of *AR* in GCs, as well as FF levels of androstenedione and testosterone, showed a highly significant positive correlation with *FSHR* mRNA expression in immature GCs [56]. A highly significant association between the gene expression of *FSHR* and *AR* in human GCs, based on an expanded dataset using a similar methodology, is shown in Fig. 7. Importantly, these findings suggest that androgen stimulation via the *AR* may enhance FSHR density and follicular sensitivity to FSH.

Androgen priming has been used for improving poor ovarian response during ovarian stimulation and has been widely used in clinical practice. While the exogenous administration of androgens, such as dehydroepiandrosterone or testosterone, has only a minor impact on follicle recruitment and reproductive outcomes, particularly when systemic androgenic effects are unwanted [57, 58]. An alternative approach may be to boost local androgen production by stimulating TC function through the administration of hCG or LH [59]. However, both strategies remain unestablished in clinical settings but a recent study in poor ovarian response patients found that increasing LH-like activity (i.e. hCG) to 10–15 IU/L over a two-month period by injecting daily low-dose hCG (250 IU) led to a 50 % increase in the number of oocytes retrieved. However, this translated to a modest absolute gain of approximately 1.5 additional oocytes per cycle [59] but emphasize that androgens likely play a role in advancing follicular development during the recruitment phase of the follicular cycle in vivo. Thus, to overcome and affect the significant concentration gradient required for androgen transport into the follicle and affecting GCs presents a challenge especially when systemic androgen administration is performed and suggests that local androgen production may be more likely to generate clinically viable options [58, 59].

These clinical data suggests that androgens locally appear to augment follicular recruitment, while systemic administration appears to have a modest effect of follicular growth, supporting that stimulating TC androgen production in the vicinity of the follicles potentially in synergy with inhibin-B is a valid mechanism operating in vivo.

### Effect of FSH on theca cell androgen production and granulosa cell oestradiol synthesis

In the ovaries, the classical concept on androgens production involves proper stimulation of TC by LH and/or hCG, the only follicular cell type expressing the key enzyme CYP17A1. The LH receptor (LHR) is constitutively expressed on TCs, and androgen production takes place already in relatively small antral follicles (Fig. 5) in which AR in GCs are also expressed [40, 42, 52, 56, 60]. FSH acts indirectly on TCs by stimulation of GCs to produce various growth factors, including inhibin-B, inhibin-A, and insulin-like growth factors (IGFs), which function as paracrine enhancers of LH-induced androgen synthesis in TCs [61]. In fact, LH has a limited stimulatory effect on human TC androgen production on its own in vitro and the presence of inhibin’s and/or IGFs is required to significantly enhances androstenedione synthesis [61, 62]. Notably, LH and IGF1 synergize to increase androgen production at least 40-fold compared to controls, and this effect was further amplified tenfold in the presence of both IGF and inhibin [62]. The concentrations of growth factors functioning as paracrine factors used in these early in vitro studies were highly supraphysiological compared to circulating levels. For instance, inhibin-B concentrations in circulation are approximately 0.05 ng/ml [63], whereas the maximal concentration tested in culture was 100 ng/ml—approximately 2000 times higher than physiological levels [62]. However, more recent studies have shown that the combined concentration of inhibin-B and inhibin-A in preovulatory follicles during the ovulatory process can exceed 500 ng/ml [64]. This suggests that inhibin levels within follicles reach extraordinarily high concentrations, indicating that TCs in vivo are likely to be exposed to high concentrations.

**Fig. 8:**
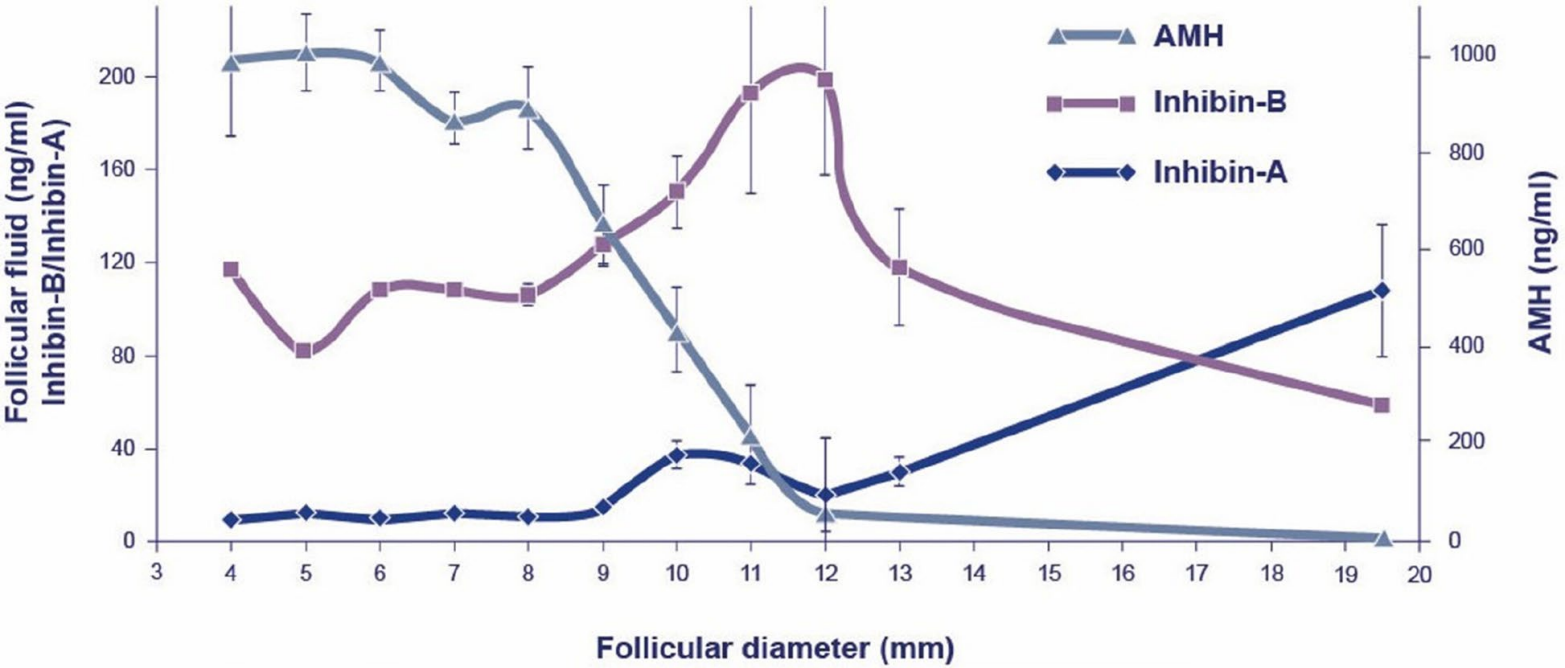
Concentrations of inhibin-B, inhibin-A, and Anti Müllerian Hormone in human follicular fluid from women in their natural cycle throughout the follicular phase. For comparison, follicular fluid collected during final follicular maturation in women undergoing ovarian stimulation shows peak concentrations of inhibin B and inhibin A at approximately 400 ng/mL and 300 ng/mL, respectively (data not shown) [64]

Further evidence supporting the role of inhibin’s in androgen production comes from studies analysing FF from normal human small antral follicles. These studies found significant positive correlations between inhibin-B concentrations and both androstenedione and testosterone, as well as strong associations between GC gene expression of *FSHR*, *LHR*, and *CYP19A1* and corresponding FF inhibin-B concentrations [10] (Table 1). In fact, inhibin-B shows a peak in FF just around the time of follicular selection (Fig. 8).

**Table 1:**
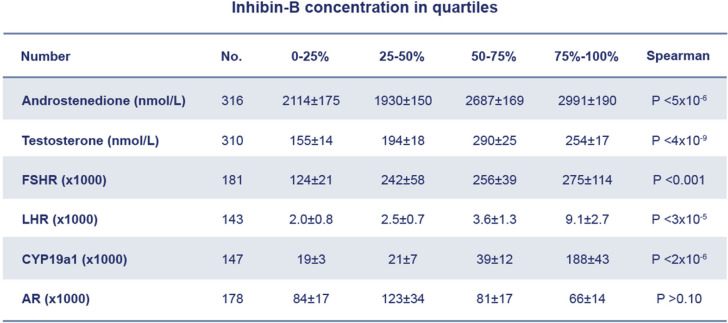
Levels of Inhibin-B in follicular fluid from small antral follicles in association to levels of androgens and gene expression in corresponding GCs; FSHR: FSH receptor, LHR: LH receptor, CYP19A1: Cytochrome P450 family 19 subfamily A member, AR: Androgen receptor [10]

Collectively, these findings suggest that in vivo, FSH-induced inhibin-B production (and likely IGF’s) in GCs plays a key role in enhancing TC androgen synthesis, highlighting the intricate regulatory mechanisms governing ovarian steroidogenesis.

Furthermore, FSH induces the expression of the *CYP19A1* gene in GCs, ensuring the aromatization of androgens into oestrogens. Notably, this expression remains limited during the first half of the follicular phase, when acidic FSH isoforms predominate, whereas CYP19A1 expression increases following follicular selection [42]. The minimal oestradiol synthesis in the early follicular phase is also likely influenced by the exceptionally high intrafollicular anti müllerian hormone (AMH) concentrations, which reach approximately 1000 ng/ml (Fig. 7) [65]. AMH has been shown to suppress CYP19A1 activity and is strongly negatively correlated with intrafollicular oestradiol levels [65–67]. At follicular selection, AMH concentrations in FF decline at least 100-fold to below 10 ng/ml, remaining low until ovulation, thereby lifting the inhibitory effect on oestradiol synthesis (Fig. 8). Consequently, oestradiol concentrations rise significantly during the preovulatory phase of follicular development.

### Effect of FSH isoforms on follicular recruitment and inhibin-B secretion in a clinical setting

Although FSH is well-known for inducing inhibin-B synthesis in human GCs, there is limited data on the differential effects of FSH isoforms on inhibin-B synthesis in a clinical setting. However, one study addressing this issue was published as an abstract for the 2007 ESHRE annual meeting [68]. The objective of the study was to compare the pharmacokinetics and pharmacodynamics of two pools of recombinant FSH isoforms purified from (rFSHα, Merck KGaA, Darmstadt, Germany) a registered recombinant FSH preparation, in non-pregnant healthy female volunteers. The two isoform pools prepared for clinical use consisted of a more acidic pool (pI 3.9–4.6) resembling that of menopausal gonadotropin preparations and a less acidic, basic pool (pI 4.2–5.1) resembling that of recombinant gonadotropin preparations [31]. Potency was determined using the Steelman-Poley assay, with 150 IU of FSH corresponding to 9 mg in the acidic isoform group and 24 mg in the less acidic isoform group. Thirty-two voluntary women underwent GnRH agonist down-regulation, and upon achieving FSH levels <4 IU/L, they were randomly assigned to receive 150 IU of FSH daily for seven days. Concentrations of oestradiol and inhibin-B were monitored daily during FSH stimulation. Additionally, ultrasound measurements were conducted before FSH administration, on days 2, 4, and 6 during FSH administration, and on days 8, 10, and 13 during follow-up to assess follicular numbers, diameters and volumes.

The two groups were similar in demographics, including age (mean: 25 years, range: 18-30) and BMI (mean: 22, range: 19-25). During FSH administration FSH concentrations were consistently higher in the group receiving basic isoforms compared to the more acidic isoforms, with an average exposure approximately 40 % higher in the less acidic isoform group. This likely reflect that more than 250 % FSH mass were administered to the group receiving less acidic FSH isoforms but with a half-life that was significantly longer in the group receiving more acidic isoforms.

Participants receiving more acidic FSH isoforms developed significantly higher number of small follicles (<10 mm in diameter) on day 8 and a significantly higher number of medium follicles (11-15 mm in diameter) on day 10 of stimulation.

Interestingly, the area under the curve for serum inhibin-B concentrations was significantly higher in the group receiving more acidic FSH isoforms compared to those receiving more basic isoforms. From day 4 after initiating FSH administration, the group receiving acidic isoforms showed markedly higher inhibin-B concentrations, which were approximately 300 % higher on days 6, 7, and 8 following initiation of FSH administration, while oestradiol concentrations remained similar between the two groups [68].

The authors concluded that administration of a more acidic pool of FSH isoforms resulted in increased follicular development and a significantly higher follicle growth rate compared to the group receiving less acidic FSH isoforms. They emphasized that the type of FSH isoform strongly influences follicle development. Interestingly, significantly higher concentrations of inhibin-B were observed with the administration of more acidic isoforms compared to the less acidic pool thereby confirming results observed in rat GCs [21].

This clinical study is the first to directly demonstrate an association between stimulation with acidic FSH isoforms and significantly increased number of follicles being recruited simultaneously with significantly increased levels of inhibin-B being synthesized in normal women in a setting similar to ovarian stimulation in ART treatments. This study provides data of interest but has unfortunately not been published in a peer-reviewed paper.

### Effects of LH during the follicular phase of the menstrual cycle

Luteinizing hormone levels remain relatively stable at approximately 4–6 IU/L throughout the follicular phase of the menstrual cycle [7]. While LHR expression on theca cells is constitutive and essential for androgen synthesis, LHR expression on GCs is initially low in the early follicular phase. As the dominant follicle is selected, LHR expression on GCs gradually increases [60]. In the later follicular phase, LH stimulates CYP19A1 and hydroxy-steroid-dehydorgenease3B2 (HSD3B2) expression in GCs and influences steroidogenesis [69, 70]. Several clinical studies have demonstrated that both endogenous and exogenous LH-like activity can enhance oestradiol production [71, 72]. However, it remains unclear whether different FSH isoforms differentially regulate LHR expression on GCs, and whether the supraphysiological FSH concentrations and different mixes of FSH isoforms used in ovarian stimulation protocols further augment LHR expression compared to other products and to the natural cycle. Although LH-like activity is widely recognized to influence folliculogenesis during the follicular phase, the precise mechanisms involved are still only partially understood.

## Discussion

### Implications of FSH isoforms and LH-like activity for follicular development during the first and second half of the follicular phase

This review presents a refined understanding of follicular growth by conceptually dividing the follicular phase into two distinct hormonal environments. In the first half, follicular recruitment and early growth are predominantly regulated by rising FSH levels, particularly acidic FSH isoforms, which promote androgen production and support pre-selection follicle development. In contrast, the second half of the follicular phase is characterized by the emergence of less acidic FSH isoforms in parallel to increasing oestradiol concentrations and increasing LH activity, which together support follicular dominance, final maturation, and preparation for ovulation.

#### During the first half

1. Low circulating levels of oestradiol secures the release of predominantly less acidic FSH isoform from the pituitary. This is confirmed clinically with good evidence.
2. Clinical data suggests that acidic FSH isoforms recruit significant more small antral follicles into growth up to at least 11-15 mm in diameter. This information is not widely acknowledged and is based on a single clinical study, and needs further clinical validation.
3. Animal and clinical data shows that acidic FSH isoforms stimulate GCs of small antral follicles (corresponding to the first half of the follicular phase) to synthesize more inhibin-B than less acidic isoforms. Direct clinical data derives from a single study and some animal data supports this notion. However, this constitutes a new hypothesis for recruitment of human follicles but requires more robust clinical data to be substantiated.
4. In vitro and in vivo data suggests that inhibin-B (and potentially inhibin-A) plays an important role in augmenting production of androgens and oestradiol, by acting in synergy with LH-inducing TC androgen production. This is supported by the exceptionally high concentrations of inhibin’s in FF from hSAF’s and strong associations to FF androgen levels, but there is limited clinical data, which needs to be expanded.
5. The high intrafollicular concentrations of androgens act in concert with GCs expression of AR to augment FSHR expression making follicles more sensitive to FSH. Robust associations between *FSHR* and *AR* are available but only primate and limited clinical data suggests that local androgen priming enhances number of recruited follicles.
6. The ability of FSH to induce expression of CYP19A1 in GCs securing oestradiol synthesis is limited by the high intrafollicular concentrations of AMH, whereas the acidic and less acidic FSH isoforms in clinical setting appear to result in similar concentrations of oestradiol. In vitro data in humans support this notion, but limited in vivo data is available.

#### During the second half one follicle is selected

1. The sharp decline in intrafollicular levels of AMH around follicular selection for dominance allow for FSH induced CYP19A1 expression resulting in gradually augmented oestradiol synthesis, which increases significantly around 3-4 days prior to the mid-cycle peak of gonadotropins causing more less acidic isoforms to be released. There is solid data to suggests this reflects in vivo conditions.
2. Less acidic isoforms are more short-lived but more potent than acidic isoforms in inducing for instance oestradiol synthesis. Good clinical evidence is available.
3. Expression of LHRs becomes upregulated in GCs and a combined action of FSH and LH stimulates growth and development of the selected follicle. There is some in vitro evidence for the effect of FSH on LHR expression.
4. Follicular fluid levels of inhibin-B peak around the time of selection for dominance but remains low until ovulation during which a peak of inhibin-B and inhibin-A is observed. This suggests that androgen production and FSHR expression is maintained/augmented while FSH concentrations have started to decline as follicular selection takes place. There is good clinical evidence for the concentrations of inhibin’s in FF.
5. In follicle selected for dominance concentrations of androgen reflect follicular viability. If androgen concentrations remain high this likely reflects that the aromatizing capacity of the GCs are reduced indicating reduced health of the follicle, while low levels indicate better viability and an efficient conversion of androgen to oestrogens.

#### During ovarian stimulation and fertility treatment

During ovarian stimulation with exogenous gonadotropins, levels of FSH reach supraphysiological levels of on average 15-18 IU/L depending on the dose administered. In contrast, levels of LH remain lower than that of the natural cycle being around 2 IU/L or lower without exogenous added LH-like activity. This accounts for regiments including GnRH agonist or antagonists but depends on specific stimulation protocol [73]. This introduces different dynamics as compared to the natural cycle, which has not yet been thoroughly addressed in vivo*.* FSH upregulate LHR expression on GCs enabling them to sustain follicular growth in an environment of low FSH but it is unknown whether the LHR density during ovarian stimulation is augmented compared to the natural cycle and whether different FSH isoforms affect LHR expression differently. This is an area for future research.

Furthermore, in connection with the use of the GnRH-antagonist protocol during ovarian stimulation, the pituitary remains active in secreting FSH [38, 39]. The supraphysiological concentrations of oestradiol due to multiple follicles are likely to cause a predominant release of less acidic FSH isoforms, which will blend with exogenously administered FSH. This suggests that pituitary FSH isoform mixture released is likely to reflect the number of recruited follicles. Thus, the FSH isoform profile in vivo may vary from one woman to another in relation to ovarian response and oestradiol concentration in concentration.

Provided the approach to ovarian stimulation is to mimic conditions during the natural follicular phase, a hypothesis for optimizing ovarian stimulation is to leverage on acidic FSH isoforms in the first half of the follicular phase, as they appear to promote follicular growth and androgen production via increased inhibin-B secretion. Further, in the second half, it may be envisioned that the choice of FSH product could be tailored based on follicular response and oestradiol levels in connection with GnRH-antagonist cycles. High oestradiol concentrations may support sufficient endogenous release of less acidic FSH isoforms, while lower oestradiol levels and fewer follicles can justify additional administration of less acidic FSH isoforms. Therefore, defining an approach close to that of natural conditions during the second half of the follicular phase requires additional research of the impact of the different FSH isoforms and how to manage the FSH isoform profile during ovarian stimulation. This is not currently a clinical strategy but should serve as as a working hypothesis that requires clinical support in future studies.

Finally, LH or hCG exert functions on TCs throughout the follicular phase and after selection of the follicle for dominance, GCs express LHR that exert several functions including stimulation of oestradiol production. Thus, concentrations of LH or hCG should be maintained at physiological levels (i.e. 4-6 IU/L) in both the first and second half of the follicular phase to 1) secure sufficient androgen production and 2) for stimulating LHR expression on GCs securing final growth of follicles.

## Data Availability

No datasets were generated or analysed during the current study.

## Abbreviations

AMH: Anti müllerian hormone
AR: Androgen receptor
ART: Assisted Reproduction Technologies
BMI: Body Mass Index
CYP17A1: Cytochrome P450 family 17 subfamily A member 1...
CYP19A1: Cytochrome P450 family 19 subfamily A member 1...
ESHRE: European Society for Human Reproduction and Embryology
FF: Follicular fluid
FSH: Follicle stimulating hormone
FSHR: Follicle stimulating hormone receptor
GC: Granulosa cells
GnRH: Gonadotropin releasing hormone
hCG: Human chorionic gonadotropin (hCG)
HSD3B2: Hydroxy steroid dehydrogenase 3B2
IGF: Insulin-like growth factors
pI: Isoelectric point
LH: Luteinizing Hormone
LHR: Luteinizing Hormone receptor
Mw: Molecular weight
nM: nmol/L
TC: Theca cells
